# Identification of the major QTL *QPm.cas-7D* for adult plant resistance to wheat powdery mildew

**DOI:** 10.3389/fpls.2022.1042399

**Published:** 2022-10-19

**Authors:** Hong Liu, Guohao Han, Tiantian Gu, Yuli Jin, Zhipeng Shi, Lixian Xing, Hanwen Yan, Jing Wang, Chenyang Hao, Meicheng Zhao, Diaoguo An

**Affiliations:** ^1^ Center for Agricultural Resources Research, Institute of Genetics and Developmental Biology, Chinese Academy of Sciences, Shijiazhuang, China; ^2^ The National Key Facility for Crop Gene Resources and Genetic Improvement, Institute of Crop Science, Chinese Academy of Agricultural Sciences, Beijing, China; ^3^ Innovative Academy of Seed Design, Chinese Academy of Sciences, Beijing, China

**Keywords:** adult plant resistance, powdery mildew, *Pm38*, haplotypes, molecular markers, common wheat

## Abstract

Developing effective and durable host plant resistance is crucial for controlling powdery mildew, a devastating disease caused by *Blumeria graminis* f. sp. *tritici* (*Bgt*). In the present study, we dissected the genetic basis of the adult plant resistance to powdery mildew using a recombinant inbred line (RIL) composed of 176 F_9_ RILs population derived from a cross between PuBing 3228 (P3228) and susceptible cultivar Gao 8901. P3228 exhibits stable adult-plant resistance to powdery mildew in the field over consecutive years. We identified two QTLs on chromosomes 7DS (*QPm.cas-7D*) and 1AL (*QPm.cas-1A*) contributed by P3228, and one QTL on 3DS (*QPm.cas-3D*) contributed by Gao 8901, which could explain 65.44%, 3.45%, and 2.18% of the phenotypic variances, respectively. By analyzing the annotated genes in the 1.168 Mb physical interval of the major QTL *QPm.cas-7D*, we locked a previously cloned adult-plant resistance gene *Pm38* that was most probably the candidate gene of *QPm.cas-7D.* Sequence alignment analysis revealed that the candidate gene of *QPm.cas-7D* in P3228 was identical to the reported *Pm38* sequence. Two haplotypes *QPm-7D-R* and *QPm-7D-S* were identified in the whole *Pm38* genomic regions between P3228 and Gao 8901. To apply *QPm.cas-7D* in wheat breeding, we developed a kompetitive allele-specific PCR (KASP) marker *Kasp5249* that is closely linked with these haplotypes. It is worth mentioning that the *QPm-7D-R* haplotype significantly decreased TKW and underwent negative selection for higher yields in China wheat breeding. In this study, we identified a major QTL *QPm.cas-7D* and revealed the relationship between its resistance and yield, which could be beneficial for further applications in wheat disease resistance and high-yield breeding.

## Introduction

Common wheat (*Triticum aestivum*) is an important contributor to national food security and sustains one-third of humankind (IWGSC, 2018). With an estimated global population of more than nine billion over the next 30 years, wheat production is facing an approximately 70% growth challenge to meet the food demands (IWGSC, 2014). However, wheat powdery mildew, a globally epidemic wheat disease caused by the biotrophic fungus *Blumeria graminis* f. sp. *tritici* (*Bgt*), can severely reduce wheat yields and affect grain quality (Yahiaoui et al., 2004; Singh et al., 2016). In recent decades, the planting area of winter wheat in China affected annually by powdery mildew has reached 6 m ha, resulting in 300,000 tons of yield loss each year (Jia et al., 2020).

Developing effective and durable host plant resistance is crucial for controlling powdery mildew epidemics. Resistance to disease in crops is typically classified into two main patterns: qualitative resistance and quantitative resistance (Spielmeyer et al., 2005; Lillemo et al., 2008; McIntosh et al., 2019; He et al., 2021). Qualitative resistance is mostly race-specific where resistance (*R*) gene based and confers strong and life-long immunity at all stages (Kang et al., 2020; Sánchez-Martín et al., 2021). However, *Bgt* isolates have complex and highly variable virulence structures, so their constant evolution causes the constant breakdown of *R* genes, particularly in areas where *R* genes were widely used (An et al., 2019). Different from qualitative resistance, quantitative resistance which conferred by polygenes is mostly non-race-specific, of which adult plant resistance (APR) is one of the main types and exhibits effectiveness at the post-seedling stages. APR usually cannot display complete immunity, to great extent, which reduces selection pressure on pathogens (Li et al., 2014). Together, these two forms of resistance have provided the genetic basis of powdery mildew resistance in wheat.

To date, more than 100 powdery mildew (*Pm*) genes/alleles at 63 loci (*Pm1*-*Pm68*, noting that *Pm8* = *Pm17*, *Pm18* = *Pm1c*, *Pm22* = *Pm1e*, *Pm23* = *Pm4c*, and *Pm31* = *Pm21*) have been found from common wheat and its wild relatives (He et al., 2018; McIntosh et al., 2020). Most of the 68 formally designated *Pm* genes provided qualitative resistance which showed all stage resistance (ASR), only *Pm38* (Lagudah et al., 2009), *Pm39* (Lillemo et al., 2008), *Pm46* (Moore et al., 2015), and *Pm62* (Zhang et al., 2018) showed APR. Despite numerous *Pm* genes having been reported, most of them cannot be directly applied in wheat production due to unexpected linkage drag or longer breeding cycles required for genes that were from wheat relatives or landraces. For instance, the broad-spectrum powdery mildew resistance gene *Pm16* led to 15% yield loss when it was introgressed into the wheat backgrounds (Summers and Brown, 2013). In the current wheat breeding programs in China, only a few *Pm* genes have been extensively used in wheat improvement (Jin et al., 2021), which are facing huge selective pressure. It is therefore necessary for resistance durability to unceasingly identify and rationally deploy various types of *Pm* genes/alleles from various germplasm resources.

Once the effective gene was identified, the next challenge is its accurate and rapid transfer in breeding programs. Compared with the conventional breeding, marker-assisted selection (MAS) is more accurate for it also combines genotypic identification. The targeted genes could be selected or excluded in fewer generations by using the powerful diagnostic markers (Jiang et al., 2016). Therefore, cloning of target genes/loci and their tightly-linked molecular markers are key points for MAS. Recent progress in whole-genome sequencing and data-processing strategies have greatly promoted the isolation of the resistance genes. Up to now, 13 *Pm* genes, including *Pm1a* (Hewitt et al., 2021), *Pm2* (Sánchez-Martín et al., 2016), *Pm3* (Yahiaoui et al., 2004), *Pm4b* (Sánchez-Martín et al., 2021), *Pm5e* (Xie et al., 2020), *Pm8* (Hurni et al., 2013), *Pm17* (Singh et al., 2018), *Pm21* (He et al., 2018; Xing et al., 2018), *Pm24* (Lu et al., 2020), *Pm38/Yr18/Lr34/Sr57* (Krattinger et al., 2009), *Pm41* (Li et al., 2020), *Pm46/Yr46/Lr67/Sr55* (Moore et al., 2005) and *Pm60* (Zou et al., 2018), have been cloned by multiple strategies. Many diagnostic markers based on variations in the functional gene sequence have been consequently developed, such as the functional kompetitive allele-specific PCR (KASP) marker *Pm5e-KASP* for *Pm5e* (Xie et al., 2020), and *STS-Pm24* for *Pm24* (Lu et al., 2020). Those markers have no recombination with the target genes, which highlighted the extremely precise and efficient genotyping results.

PuBing3228 (P3228) is a wheat-*Agropyron cristatum* introgression line (Liu et al., 2020). It exhibits stable resistance to powdery mildew in wheat-growing regions over consecutive years, indicating that it should be a promising resource for durable powdery mildew resistance. To better clarify and use the resistance against powdery mildew in P3228, the objectives of this study were to (i) assess its resistance to powdery mildew, (ii) map the major QTL for powdery mildew resistance and predict candidate gene(s), (iii) reveal the relationship between *Pm* gene(s) and yield traits, (iv) determine the geographical distribution of the major *Pm* gene(s), and (v) develop KASP marker of the candidate gene(s) for MAS breeding.

## Materials and methods

### Plant materials and field trials

**Figure 1:**
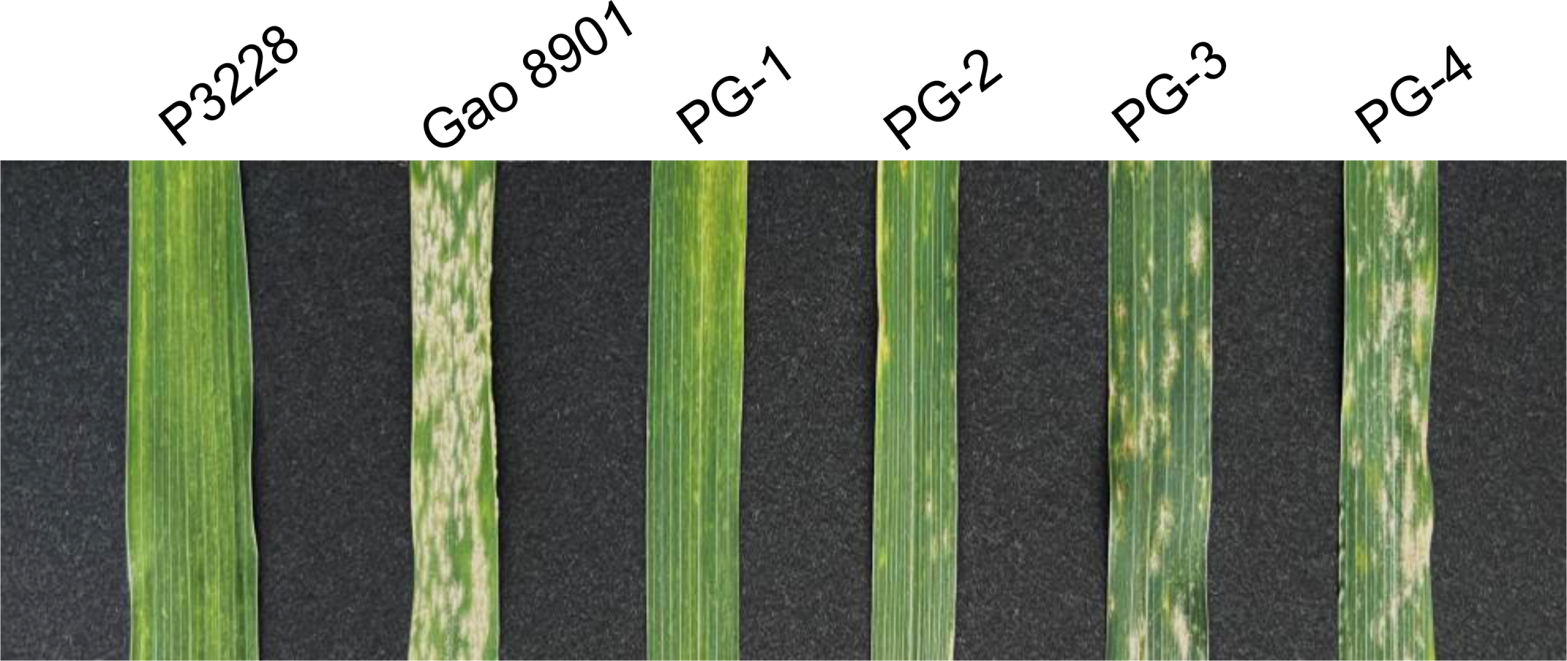
Phenotypic characterization of resistance to powdery mildew at the adult stage in two parents and some representative RILs. P3228, PuBing 3228; PG-1, PG-2, PG-3, and PG-4 are representative lines in PG-RIL population.

A mapping population composed of 176 F_9_ RILs derived from ‘P3228 × Gao 8901’ was developed by single seed descent method. The wheat line P3228 exhibited resistance to powdery mildew at the adult stage whereas Gao 8901 was highly susceptible (**Figure 1**). The 176 RILs, and two parents were grown at the Center for Agricultural Resources Research, Institute of Genetics and Developmental Biology, Chinese Academy of Sciences during 2021-2022 growing seasons (2022SJZ). Hill-drop (20 seeds per hill) were used and all trials used a randomized block design with three replicates. The water, fertilizer and other management of all field trials were carried out in accordance with local standard practices. Two natural populations including 157 landraces of the Chinese wheat mini-core collection, and 348 modern cultivars from ten ecological zones of China were used for marker screening and association analysis as previously described (Zhao et al., 2019; Liu et al., 2020).

### Phenotypic assessment of powdery mildew

Seedling stage reactions of P3228 and Gao 8901 to virulent *Bgt* isolates E09, E11, and E20 were separately tested with three replicates at a greenhouse as previously described (Qie et al., 2019). Heng 4399 was used as the susceptible control. For each line, 20 seeds were planted in rectangular trays (54 cm × 28 cm × 4 cm) with 128 wells (3 cm × 3 cm × 4 cm). After 15 days after inoculation, when sporulation was observed on the first leaf of Heng 4399, the tested plants were scored using a 0-4 scale, in which infection types (ITs) 0-2 considered resistant, while ITs 3-4 considered susceptible (Si et al., 1992).

At the jointing stage, the plants were inoculated with a mixture of *Bgt* isolates E09, E11, and E20. Adult plant reactions to powdery mildew were scored with mean maximum disease severity (MDS). When the susceptible control (Heng 4399) reached 80%, the MDS scores were calculated based on the Cobb scale (Peterson et al., 1948). According to the actual percentage of powdery mildew covered area (0-100%), the severity of infection in the secondary leaves (leaf below flag leaf) of five randomly selected plants in each hill was scored. Disease severities of five selected plants in each line were averaged to obtain a mean severity for each line. Each plant was assessed twice for confirmation.

### QTL mapping

A whole-genome genetic map of the PG-RIL population to analysis the genetics of MDS was previously constructed from Wheat 660 K SNP array data (Liu et al., 2020). QTL mapping was conducted in IciMapping v4.1 software by the inclusive composite interval mapping of additive and dominant QTL (ICIM-ADD, Meng et al., 2015). The logarithm of odds (LOD) score≥2.5 (Sun et al., 2013). The MapChart 2.2 was used to draw the genetic map (Voorrips, 2002). The QTLs were named based on McIntosh et al. (2019) and ‘cas’ represents the Chinese Academy of Sciences.

### Comparison of the identified QTLs with the known powdery mildew resistance genes

The physical position of the QTL was identified using the flanking SNP markers sequence of QTL to BLAST against the genome sequences of Chinese Spring v2.1 (Zhu et al., 2021). Candidate genes in the QTL interval was acquired based on coding sequence of Chinese Spring v2.1 and gene function annotations were manually using NCBI Non-redundant protein sequences.

### Development of KASP markers

To develop markers that can efficiently trace the powdery mildew resistance genes in P3228 in MAS, KASP marker *Kasp5249* (QPm-7D-FAM: gaaggtgaccaagttcatgctATGGGAGCATTATTTTTTTCCATCT, QPm-7D-HEX: gaaggtcggagtcaacggattATGGGAGCATTATTTTTTTCCATCA, QPm-7D-R: TGCTCATCTCTGGTATGCCATTTAA) was developed based on the distinctive insertions/deletions (InDels) in the targeted interval. KASP assays were performed in 96-well format in 5 μl mixture comprising 2.81 μl of 2 × KASP mix (LGC Genomics, UK), 1μl of DNA template, 1.11 μl of ddH_2_O and 0.08 μl of primer mixture. KASP reactions were carried out on an Applied Biosytems™ Veriti™ 96 PCR system (Thermo Fisher, USA). PCR amplification procedure was performed as previously described (Liu et al., 2020). The fluorescence value was read using FLUOstar Omega SNP (LGC Genomics, UK). The KASP genotyping results were read using KlusterCaller genotyping software (LGC Genomics, UK).

### Phenotypic evaluation of agronomic traits

Phenotypic traits of 157 landraces and 348 modern cultivars, including thousand kernel weight (TKW), kernel number per spike (KNS), spikelet number per spike (TSS), spike length (SL), effective tiller number (ETN) and plant height (PH), were investigating from plants grown in 2002 and 2005 at Luoyang, Henan province and 2010 at Shunyi, Beijing.

### Statistical analyses

The frequency distribution of powdery mildew responses and analysis of variance (ANOVA) was calculated in performed with SPSS Statistics v20.0 software (SPSS, USA). The broad-sense heritability (*H^2^*) was calculated using the QGAStation 2.0 (http://ibi.zju.edu.cn/software/qga/v2.0/indexc.htm) and the following formula *H^2^* = *VG*/*VP*; where *VG* and *VP* are the genetic variance and phenotypic variance, respectively. Two-tailed *t* test was performed with SPSS Statistics v20.0 software (SPSS, USA).

## Results

### Evaluation of powdery mildew resistance and correlation analysis

At the seedling stage, both P3228 and Gao 8901 developed abundant sporulation on the leaves with an IT 4 when inoculated with *Bgt* isolate E09, E11 and E20, respectively. At the adult plant stage, when the MDS of the susceptible control Heng 4399 ranged from 80% to 100%, the P3228 and Gao 8901 showed 1.00% and 67.67%, respectively, showing significant differences on MDS (**Table 1**). For the RIL population, the frequency distributions of MDS showed continuous variation (**Supplemental Figure 1**). The MDS scores showed broad-sense heritability (*H^2^*) at 0.63 (**Table 1**).

**Table 1:** Phenotypes of the parents and PG-RIL population in this study.

Trait	Parents		PG-RILs					
	P3228	Gao 8901	Minimum	Maximum	Mean	SD	CV(%)	*H*
MDS	1.00	66.67	0.20	100.00	46.61	35.78	76.76	0.63

MDS, maximum disease severities.

### QTL mapping

**Figure 2:**
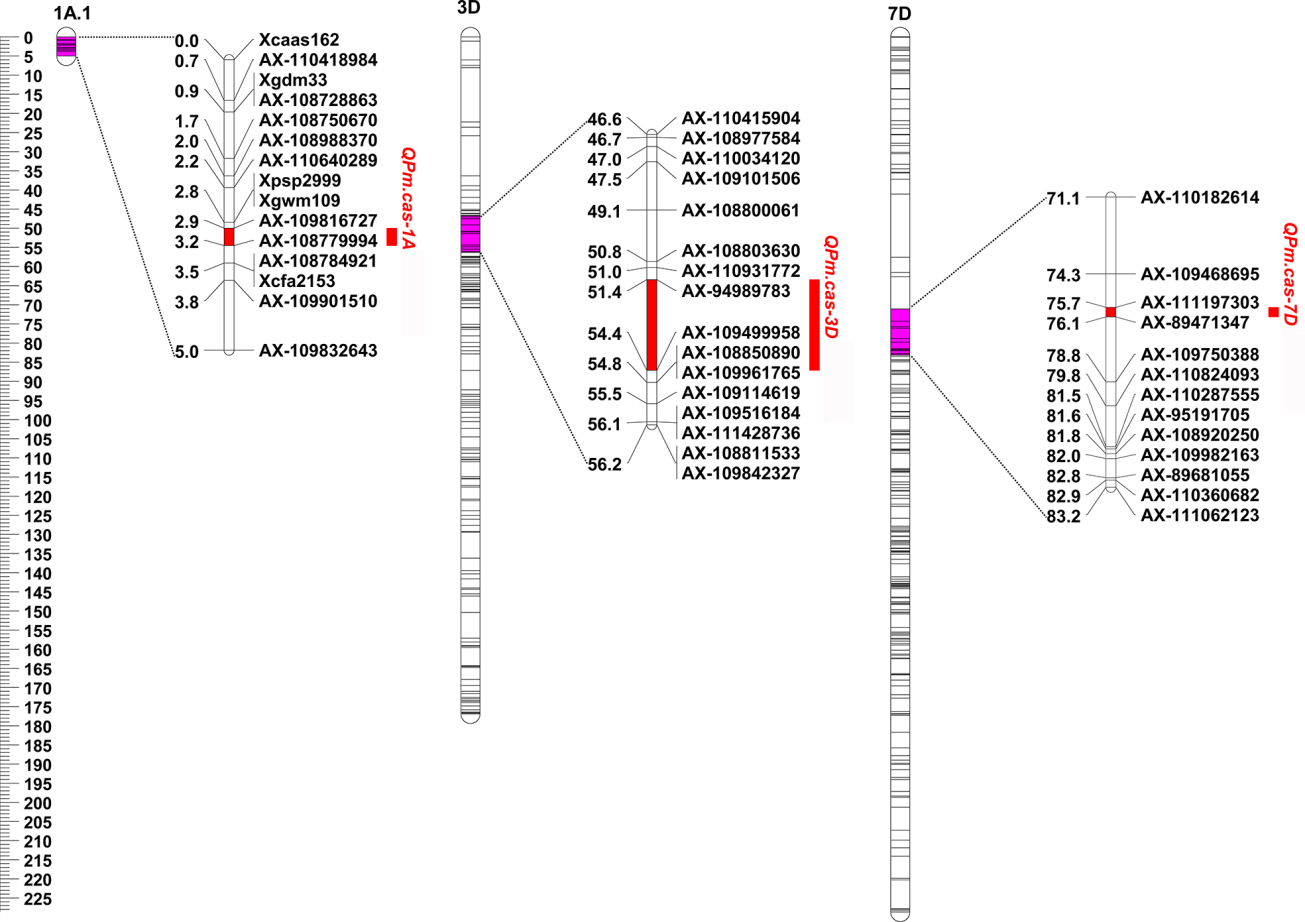
Genetic locations of QTL intervals associated with maximum disease severities detected in 2022SJZ environment. Uniform centimorgan (cM) scales are shown on the left. QTLs are indicated on the right side of each chromosome.

Based on the results of powdery mildew reaction evaluation, two QTLs from P3228 on chromosomes 1A and 7D, and one from Gao 8901 on chromosomes 3D, respectively, were detected in 2022SJZ environment (**Table 2** and **Figure 2**), and were designated as *QPm.cas-1A*, *QPm.cas-3D*, and *QPm.cas-7D*, respectively. The QTL *QPm.cas-1A* was located in the marker interval *AX-109816727*–*AX-10877999* on the short arm of chromosome 1A and explained 3.45% of the phenotypic variance with an additive effect of -10.58 (**Table 2** and **Figure 2**). The QTL *QPm.caas-3DS* was mapped on chromosome 3DS and flanked by markers *AX-94989783* and *AX-109499958*, which accounted for 2.18% of the phenotypic variance with an additive effect of 5.33 (**Table 2** and **Figure 2**). The major QTL *QPm.cas-7D* was mapped on marker interval *AX-111197303*–*AX-89471347* on the short arm of chromosome 7D and explained 65.44% of the phenotypic variance with an additive effect of -29.38 (**Table 2** and **Figure 2**).

**Table 2:** QTLs for maximum disease severities (MDS) in the PG-RIL population.

Trait	QTL	Markers Interval	Genetic Interval (cM)	Physical Interval (Mb)	LOD	PVE%	Add
MDS	*QPm.cas-1A*	*AX-109816727*– *AX-108779994*	2.932–3.173	6.64–7.63	4.75	3.45	-10.58
	*QPm.cas-3D*	*AX-94989783*– *AX-109499958*	51.373–54.408	58.18–65.88	3.13	2.18	5.33
	*QPm.cas-7D*	*AX-111197303–* *AX-89471347*	75.65–76.066	48.92–50.09	47.39	65.43	-29.38

LOD, threshold log-of-odds; PVE, phenotypic variance explained; Add, additive effect.

### Predicting of candidate gene *Pm38* for QTL *QPm.cas-7D*

**Figure 3:**
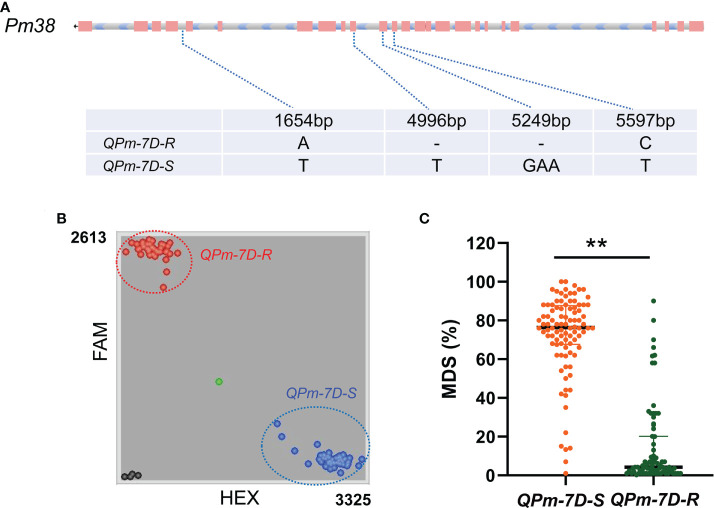
Haplotypes analysis with maximum disease severities (MDS) of *QPm.cas-7D* in PG-RIL population. **(A)** Haplotypes of *QPm.cas-7D* based on its genome regions in P3228 and Gao 8901. **(B)** Genotyping results of the diagnostic marker *Kasp5249* developed for *QPm-7D-R* and *QPm-7D-S* haplotypes in the PG-RIL population. **(C)** Haplotypes analysis with MDS of *Pm38* in the PG-RIL population. ^**^ *P* < 0.01 (two-tailed *t* test).

The peak interval of the major QTL *QPm.cas-7D* was collocated between the markers *AX-111197303* and *AX-89471347*. Combined with the physical position of Bin markers *Bin-AX-111197303* (including *AX-111197303* and *AX-89378255*) and *Bin-AX-89471347* (including *AX-89471347*) based on the Chinese Spring reference genome v2.1 (Zhu et al., 2021), the QTL *QPm.cas-7D* was mapped to the 48.917–50.085 Mb position on chromosome arm 7DS. We found 17 high confidence annotated genes in the 1.168 Mb region using Chinese Spring reference genome v2.1 (**Table 3**). Among them, *Pm38* (*TraesCS7D03G0183600*), a previously cloned adult-plant resistance gene to powdery mildew, was considered as the preferred candidate gene for *QPm.cas-7D*. Then, we analyzed the genomic sequence of *Pm38* from Gao 8901 and P3228 using two pairs of genome-specific primers as reported to amplify the gene separately from the start codon to exon 14 (ExpF1 and Cssfr6-MR1), and from exon 11 to the stop codon (Cssfr6-MF2 and Lr34-ExpR1) (Fang et al., 2020). The results showed that the *Pm38* allele in P3228 was identical to the previously reported *Pm38*. Moreover, two SNPs (A1654T and C5597T) and two InDels (1-bp InDels at 4996^th^ and 3-bp InDels at 5249^th^) were identified between P3228 and Gao 8901 in the whole *Pm38* genomic regions, which formed two haplotypes: *QPm-7D-R* (resistant haplotype) and *QPm-7D-S* (susceptible haplotype) (**Figure 3A**). Meanwhile, sequence alignment revealed that the 1-bp deletion at 4996^th^ caused frameshift mutation, resulting in a loss-of-function *Pm38* protein in Gao 8901 (**Figure 3A**).

**Table 3:** Candidate genes identified for QTL *QPm.cas-7D* with putative functions of interest and their functional annotation.

Gene Name	Blast-hit-accession	Description
*TraesCS7D03G0183500*	tr|B9R9W6|B9R9W6_RICCO	Sugar transporter
*TraesCS7D03G0183600*	tr|W0TSU1|W0TSU1_ACAMN	Pleiotropic drug resistance ABC transporter
*TraesCS7D03G0183700*	tr|C0JSA9|C0JSA9_WHEAT	Cytochrome P450
*TraesCS7D03G0183800*	tr|B8XSM8|B8XSM8_WHEAT	Lectin receptor kinase
*TraesCS7D03G0183900*	tr|B8XSM9|B8XSM9_WHEAT	Lectin receptor kinase
*TraesCS7D03G0184100*	tr|B8XSN0|B8XSN0_WHEAT	Cytochrome P450
*TraesCS7D03G0184300*	AT1G65040.4	RING/U-box superfamily protein
*TraesCS7D03G0184400*	tr|A0A165XS50|A0A165XS50_DAUCA	UDP-glycosyltransferase
*TraesCS7D03G0184600*	tr|A0A165XS50|A0A165XS50_DAUCA	UDP-glycosyltransferase
*TraesCS7D03G0184900*	AT5G19400.5	Telomerase activating protein Est1
*TraesCS7D03G0185400*	tr|A0A061DYZ5|A0A061DYZ5_THECC	Ubiquitin-conjugating enzyme 23 isoform 1
*TraesCS7D03G0185500*	tr|A0A061DYZ5|A0A061DYZ5_THECC	Ubiquitin-conjugating enzyme 23 isoform 1
*TraesCS7D03G0185700*	tr|K7TR03|K7TR03_MAIZE	Basal layer antifungal peptide
*TraesCS7D03G0185800*	AT4G22640.2	Bifunctional inhibitor/lipid-transfer protein/seed storage 2S albumin superfamily protein
*TraesCS7D03G0186600*	tr|A0A0K9NQW8|A0A0K9NQW8_ZOSMR	Tyrosine-tRNA ligase
*TraesCS7D03G0187100*	tr|A0A103XL20|A0A103XL20_CYN	Elongation factor 4
*TraesCS7D03G0187200*	tr|A0A059PZI7|A0A059PZI7_9POAL	Carboxypeptidase

### Development of KASP markers and analysis for *Pm38* alleles

Based on the 3-bp InDel on the *Pm38*- homologous sequence between P3228 and Gao 8901, we developed a KASP marker *Kasp5249* (**Figure 3B**). After screening the PG-RIL population using marker *Kasp5249*, a two-tailed *t* test was performed between the InDel of *Kasp5249* and MDS. The results showed that *Kasp5249* was significantly correlated with MDS in the PG-RIL population (**Figure 3C**). These results further demonstrated that the candidate gene for *QPm.cas-7D* was most likely *Pm38*.

### Association analysis of *QPm-7D-R* haplotype with yield-related traits in common wheat

**Figure 4:**
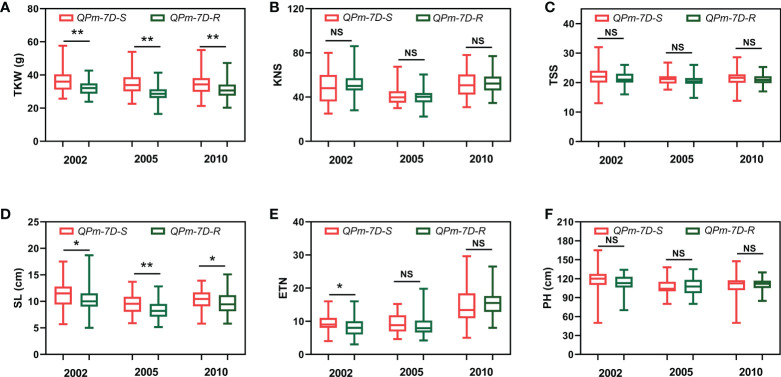
Haplotypes analysis with agronomic traits of *QPm.cas-7D* in the landraces of the Chinese wheat mini-core collection. Comparison analysis of *QPm.cas-7D* haplotypes with the TKW **(A)**, KNS **(B)**, TSS **(C)**, SL **(D)**, ETN **(E)**, and PH **(F)** of the landraces of the Chinese wheat mini-core collection in three environments. ^**^ *P* < 0.01 and ^*^ *P* < 0.05 (two-tailed *t* test) indicates a significant difference to the two haplotypes. NS, no significant difference.

**Figure 5:**
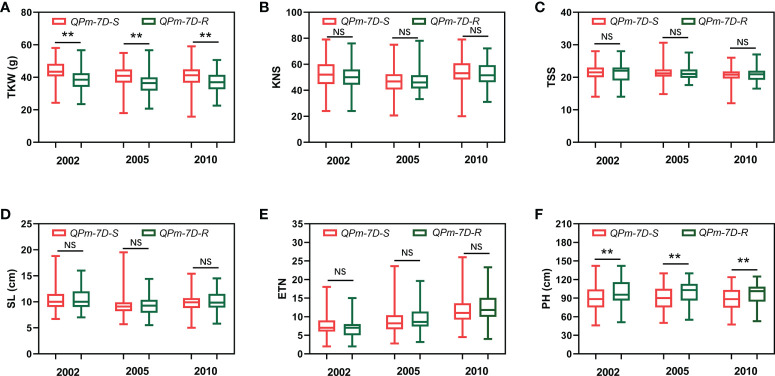
Haplotypes analysis with agronomic traits of *QPm.cas-7D* in the Chinese modern cultivars. Comparison analysis of *QPm.cas-7D* haplotypes with the TKW **(A)**, KNS **(B)**, TSS **(C)**, SL **(D)**, ETN **(E)**, and PH **(F)** of the Chinese modern cultivars in three environments. ^**^ *P* < 0.01 and ^*^ *P* < 0.05 (two-tailed *t* test) indicates a significant difference to the two haplotypes. NS, no significant difference.

After screening 157 landraces of the Chinese wheat mini-core collection and 348 Chinese modern cultivars using the diagnostic marker *Kasp5249*, we performed haplotype association analysis of six agronomic traits (TKW, KNS, TSS, SL, ETN, PH) in multiple environments. The resistance haplotype *QPm-7D-R* was significantly correlated with TKW and SL in the 157 landraces of the Chinese wheat mini-core collection (**Figures 4A-F**). The mean TKW of *QPm-7D-R* plants was significantly lower than those of the *QPm-7D-S* plants (4.47 g lower in 2002, 5.71 g lower in 2005, and 3.55 g lower in 2010) (**Figure 4A**). Similar results were found in 348 Chinese modern cultivars. Significant differences were also detected in TKW and PH between *QPm-7D-R* and *QPm-7D-S* haplotypes (**Figures 5A-F**). The mean TKW of *QPm-7D-R* plants in three environments was also significantly lower than that of *QPm-7D-S* plants (5.28 g lower in 2002, 4.58 g lower in 2005, and 3.49 g lower in 2010) (**Figure 5A**). In summary, the above results indicate that *QPm-7D-R* is the lower TKW haplotype than *QPm-7D-S.*

### *QPm.cas-7D-R* haplotype underwent negative selection during Chinese wheat breeding

**Figure 6:**
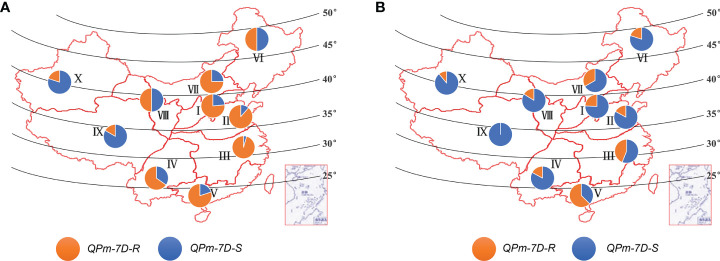
Geographic distribution of the *QPm.cas-7D* haplotypes among ten Chinese ecological regions. Distribution of *QPm.cas-7D* haplotypes in landraces **(A)** and modern cultivars **(B)** among ten Chinese ecological regions. I, northern winter wheat region; II, Yellow and Huai River valley winter wheat region; III, low and middle Yangtze River valley winter wheat region; IV, southwestern winter wheat region; V, southern winter wheat region; VI, northeastern spring wheat region; VII, northern spring wheat region; VIII, northwestern spring wheat region; IX, Qinghai–Tibet spring–winter wheat region; X, Xinjiang winter–spring wheat region.

The geographic distribution of the *QPm-7D-R* and *QPm-7D-S* haplotypes was evaluated in both landraces and modern cultivars from ten ecological zones of China. The frequency of the *QPm-7D-R* haplotype declined in the modern cultivars relative to the landraces in the major Chinese production zones (**Figures 6A, B**). By contrast, the frequency of the *QPm-7D-S* haplotype with high TKW was increased during the transition from landraces to modern cultivars (**Figures 6A, B**). These results suggested that *QPm-7D-R* underwent negative selection in China wheat breeding for higher yields.

### Discussion

P3228 is a valuable wheat germplasm line carrying multiple and stable QTLs related to yield traits (Wang et al., 2011; Liu et al., 2020). In the present study, we found that P3228 was susceptible to *Bgt* isolates E09, E11, and E20 at the seedling stage but highly resistant to a mixture of those *Bgt* isolates at the adult plant stage, suggesting that P3228 conferred APR against powdery mildew (**Figure 1**). Subsequently, using a RIL population of ‘P3228 × Gao 8901’, we identified a major QTL *QPm.cas-7D* contributed by P3228 in an interval flanked by *AX-111197303*–*AX-89471347* on the short arm of chromosome 7D, which explained 65.44% of the phenotypic variance with an additive effect of -29.38 (**Table 2** and **Figure 2**). Meanwhile, a minor QTL *QPm.cas-1A* also from P3228 was detected in the marker interval *AX-109816727*–*AX-10877999* on the short arm of chromosome 1A and explained 3.45% of the phenotypic variance with an additive effect of -10.58 (**Table 2** and **Figure 2**). *Pm38* (*TraesCS7D03G0183600*), a previously cloned *Pm* gene conferring APR against powdery mildew (Krattinger et al., 2009) was included in the targeted physical interval of the major QTL *QPm.cas-7D*. Sequencing showed that P3228 had an identical sequence to the reported *Pm38* (Krattinger et al., 2009), while Gao 8901 had two different SNPs and two InDels in the genomic regions, resulting a frameshift mutation, the findings indicated that *QPm.cas-7D* was most likely *Pm38* (**Figure 3A**). Of course, we cannot completely exclude the possibility that *QPm.cas-7D* might be one of the other 17 candidate genes that cooperated with *Pm38* in the targeted physical interval. Further fine mapping and functional validation of the candidate genes should be performed to confirm the *Pm38* as the caused gene for *QPm.cas-7D* in the future.

Due to the ease of selection and phenotypic evaluation, most disease resistance studies have focused on ASR genes, which are known as qualitative or race-specific resistance genes (Wu et al., 2022). However, overuse of single race-specific resistance genes can lead to the rapid evolution of new virulent *Bgt* races and consequent massive economic losses (Singh et al., 2016). In this case, developing durable resistance conferred by APR genes is emphasized. Many APR genes appear to provide broad-spectrum resistance to one or multiple diseases at the same locus, such as *Pm38/Lr34/Yr18/Sr57* (Krattinger et al., 2009), *Pm39/Lr46/Yr29/Sr58* (Singh et al., 2013), *Pm46/Lr67/Yr46/Sr55* (Moore et al., 2015), and *Pmx/Lr27/Yr30/Sr2* (Mago et al., 2011). Previous studies revealed that the pyramiding of multiple APR genes enabled near-immunity of the plants (Singh and Trethowan, 2007). Therefore, the QTL for powdery mildew resistance identified in P3228 could enrich the available wheat genetic resources in breeding for durable and multiple resistance.

The balance of resistance and yield is the main concern for breeders when using a resistance gene in wheat breeding programs, but often, disease resistance is at the expense of some agronomic traits and reduces plant adaptation (Deng et al., 2017; Han et al., 2022; Mu et al., 2022). For example, *mildew resistance locus O* (*MLO*), is a durable and broad-spectrum resistance to powdery mildew in various plant species including common wheat, however, it also leads to growth penalties and yield losses, thereby limiting its widespread use in wheat breeding (Li et al., 2022). So, investigation of the corresponding yield traits tends to be an important index for evaluating the use of resistance gene(s). Recent research shows that *Pm5e* has no yield penalty by investigating the agronomic performance in a pair of near-isogenic lines H962R with *Pm5e* and H962S without *Pm5e* (Qiu et al., 2022). In this study, we found that *QPm.cas-7D* significantly decreased TKW in the 157 landraces of the Chinese wheat mini-core collection and 348 Chinese modern cultivars (**Figures 4A-F** and **Figures 5A-F**). An additional interesting phenomenon was also observed that the frequency of *QPm.cas-7D* in wheat landraces was higher than in breeding lines, which might attribute to the artificial selection of high TKW trait in wheat major production regions. Thus, *QPm.cas-7D* could be designed to transfer into the various wheat cultivars exhibiting desirable performance on TKW.

To better transfer *QPm.cas-7D* in MAS, we developed a KASP marker *Kasp5249* (**Figure 3B**) based on the differences in the *Pm38*- homologous sequence between P3228 and Gao 8901. It is worth mentioning that *Kasp5249* was developed according to the 3-bp deletion that causes the loss of protein function, which could also be used as the functional marker of *Pm38*. We believe that after the suitable selection for resistance and agronomic performance, *QPm.cas-7D* will release its full potential in wheat breeding programs.

## Conclusion

We performed QTL analysis using the PG-RIL population for MDS, and three QTLs *QPm.cas-1A*, *QPm.cas-3D*, and *QPm.cas-7D* were identified in 2022SJZ environment (**Table 2** and **Figure 2**). Notably, the major QTL *QPm.cas-7D* contributed by P3228, could explain 65.44% of the phenotypic variances (**Table 2**). Furthermore, the QTL *QPm.cas-7D* was delimited to the physical interval of approximately 1.168 Mb, and *Pm38* was considered as the candidate gene (**Table 3**). Based on a 3-bp InDel of *Pm38* genomic sequence between the two parents, a KASP marker *Kasp5249* of *Pm38* allele was developed and verified by PG-RIL population (**Figures 3B, C**). Furthermore, the *QPm-7D-R* haplotype significantly decreased TKW and underwent negative selection in China wheat breeding for higher yields (**Figures 4A-F** and **Figures 5A-F**). Our finding identified a major QTL *QPm.cas-7D* and analyzed its effects for yield-related traits, which could be helpful in improving wheat disease resistance and high-yield breeding.-

## Data availability statement

The original contributions presented in the study are included in the article/**Supplementary Material**. Further inquiries can be directed to the corresponding authors.

## Author contributions

DA and MZ conceived the study. TG, YJ, LX, HY, JW, and CH evaluated the phenotype. HL, GH and ZS carried out QTL mapping, predicted candidate gene, and developed the KASP markers. HL, GH and TG analyzed the data and wrote the manuscript. DA and MZ supervised and revised the writing of the article. All authors approved the final manuscript. All authors contributed to the article and approved the submitted version.

## Funding

This research was supported by the Strategic Priority Research Program of the Chinese Academy of Sciences (XDA24030102), the National Natural Science Foundation of China (32101686), and the Hebei Province Key Research and Development Program (22326306D). The funding bodies were not involved in the design of the study, and collection, analysis, and interpretation of data, and manuscript writing.

## Acknowledgments

The authors are grateful to Prof. Xueyong Zhang for providing 157 landraces of the Chinese wheat mini-core collection and 348 Chinese modern cultivars.

## Conflict of interests

The reviewer JL declared a shared affiliation with the author CH to the handling editor at the time of review.

The remaining authors declare that the research was conducted in the absence of any commercial or financial relationships that could be construed as a potential conflict of interest.

## Publisher’s note

All claims expressed in this article are solely those of the authors and do not necessarily represent those of their affiliated organizations, or those of the publisher, the editors and the reviewers. Any product that may be evaluated in this article, or claim that may be made by its manufacturer, is not guaranteed or endorsed by the publisher.
