# Supplementary figures and images for: Identification of the major QTL QPm.cas-7D for adult plant resistance to wheat powdery mildew
Source: Front Plant Sci. 2022 Oct 19;13:1042399. doi: 10.3389/fpls.2022.1042399 (PMC9627495; doi:10.3389/fpls.2022.1042399)

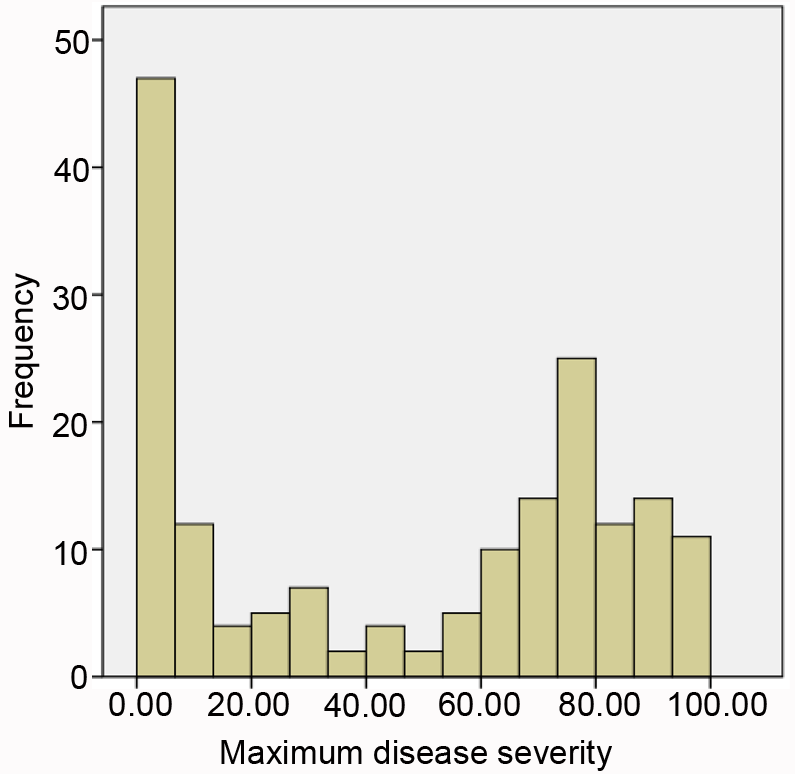


**Supplemental Figure 1** Frequency distribution of maximum in RIL population in 2022SJZ environment.

Supplement: Supplementary file 1 [file DataSheet_1.docx]
